# Whole-Genome Array CGH Evaluation for Replacing Prenatal Karyotyping in Hong Kong

**DOI:** 10.1371/journal.pone.0087988

**Published:** 2014-02-05

**Authors:** Anita S. Y. Kan, Elizabeth T. Lau, W. F. Tang, Sario S. Y. Chan, Simon C. K. Ding, Kelvin Y. K. Chan, C. P. Lee, Pui Wah Hui, Brian H. Y. Chung, K. Y. Leung, Teresa Ma, Wing C. Leung, Mary H. Y. Tang

**Affiliations:** 1 Department of Obstetrics and Gynaecology, Li Ka Shing Faculty of Medicine, The University of Hong Kong, Hong Kong Special Administrative Region, China; 2 Department of Obstetrics and Gynaecology, Queen Mary Hospital, Hong Kong Special Administrative Region, China; 3 Department of Paediatrics and Adolescent Medicine, Queen Mary Hospital, Li Ka Shing Faculty of Medicine, The University of Hong Kong, Hong Kong Special Administrative Region, China; 4 Department of Obstetrics and Gynaecology, Queen Elizabeth Hospital, Hong Kong Special Administrative Region, China; 5 Department of Obstetrics and Gynaecology, Kwong Wah Hospital, Hong Kong Special Administrative Region, China; University Medical Center Hamburg-Eppendorf, Germany

## Abstract

**Objective:**

To evaluate the effectiveness of whole-genome array comparative genomic hybridization (aCGH) in prenatal diagnosis in Hong Kong.

**Methods:**

Array CGH was performed on 220 samples recruited prospectively as the first-tier test study. In addition 150 prenatal samples with abnormal fetal ultrasound findings found to have normal karyotypes were analyzed as a ‘further-test’ study using NimbleGen CGX-135K oligonucleotide arrays.

**Results:**

Array CGH findings were concordant with conventional cytogenetic results with the exception of one case of triploidy. It was found in the first-tier test study that aCGH detected 20% (44/220) clinically significant copy number variants (CNV), of which 21 were common aneuploidies and 23 had other chromosomal imbalances. There were 3.2% (7/220) samples with CNVs detected by aCGH but not by conventional cytogenetics. In the ‘further-test’ study, the additional diagnostic yield of detecting chromosome imbalance was 6% (9/150). The overall detection for CNVs of unclear clinical significance was 2.7% (10/370) with 0.9% found to be de novo. Eleven loci of common CNVs were found in the local population.

**Conclusion:**

Whole-genome aCGH offered a higher resolution diagnostic capacity than conventional karyotyping for prenatal diagnosis either as a first-tier test or as a ‘further-test’ for pregnancies with fetal ultrasound anomalies. We propose replacing conventional cytogenetics with aCGH for all pregnancies undergoing invasive diagnostic procedures after excluding common aneuploidies and triploidies by quantitative fluorescent PCR. Conventional cytogenetics can be reserved for visualization of clinically significant CNVs.

## Introduction

Conventional cytogenetics has been the gold standard for detecting chromosomal abnormalities in prenatal diagnosis. It enables the examination of genome-wide numerical and structural abnormalities at microscopic level, and can achieve a resolution of 5–10 Mb [1]. However, the method is labour intensive, with a turn-around time of 14 to 21 days. Various molecular cytogenetic techniques, such as Quantitative Fluorescent Polymerase Chain Reaction (QF-PCR) [2], [3] and Fluorescent In Situ Hybridization (FISH) technology, could complement the detection of chromosomal abnormalities and offer faster turn-around times. However, these methods are targeted to detect specific chromosomal abnormalities and are dependent on the chromosomal probe used. In contrast, whole-genome array comparative hybridization (aCGH) not only provides high resolution detection of genomic alterations, but also allows refinement of breakpoints on chromosome rearrangements.

Chromosomal microarray to assess DNA copy number variations has been suggested as the first-tier clinical diagnostic test in the postnatal setting for individuals with developmental disabilities or congenital anomalies because of an increased diagnostic yield of 12 to 15% compared to conventional karyotyping [4], [5]. The clinical utility of aCGH in the prenatal setting has been demonstrated in systematic reviews [6]–[8], a large scale prospective randomized controlled trial [9] and also in recent research [10]. The major challenge for the large-scale implementation of these techniques appears to lie in interpretation of the results [11]. Thus genetic counseling and ethical issues [12], [13] are significant in offering whole-genome aCGH in prenatal diagnosis. Essentially, there is a need for consensus in determining which patient groups should be offered in routine prenatal practice, and international guidance on interpretation and reporting of copy number variations for prenatal population [11]. This evaluation study demonstrates the advantages and disadvantages of aCGH and argues that whole-genome oligonucleotide aCGH is able to replace conventional cytogenetics in prenatal diagnosis in the local population of Hong Kong.

In this study, the use of aCGH for prenatal diagnosis is evaluated in two models: (1) as a first-tier test and, (2) as a ‘further-test’ analyzing prenatal samples of patients with abnormal fetal ultrasound findings and normal karyotypes. Results from aCGH are compared with those from conventional cytogenetics in order to determine the concordance of results and the additional diagnostic yield of aCGH over karyotyping.

## Methods

### Patients and samples

**Figure 1:**
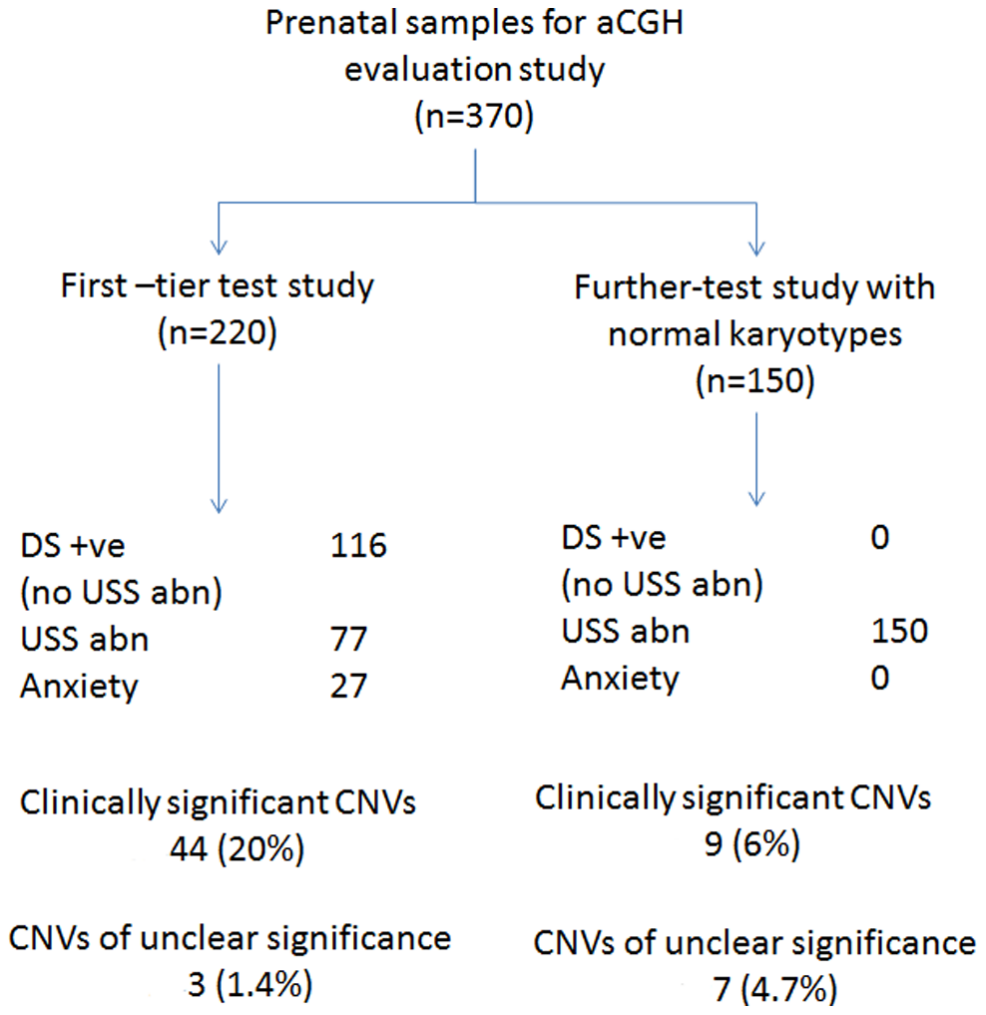
A schematic diagram showing the indications for recruitment to the study and CNVs detected in the evaluation study. The samples were subjected to first-tier test and ‘further-test’, with the clinical indications of testing and findings stated. aCGH, array CGH; CNVs, copy number variants; n, number of samples; DS +ve, Down syndrome screening positive; USS abn, ultrasound abnormality; Anxiety: maternal anxiety. Details on the clinically significant CNVs and CNVs of uncertain clinical significance are listed in Tables 1, 2, 3, 4, 5, 6.

Approval was granted by the Institutional Review Board, University of Hong Kong/Hospital Authority, Hong Kong for the study to be conducted within 3 hospitals: Tsan Yuk Hospital, Queen Elizabeth Hospital, and Kwong Wah Hospital. Written informed consent was obtained from participants, who were recruited between January 2011 and November 2012. Informed consent and counseling on the benefits and limitations of the test, test methodology, reporting time, and possible test results (clinically significant, unclear clinical significance, benign) and outcomes of the investigation were explained to the participants by medical staff. Parental blood samples were obtained at the time of consent in case information on inheritance of CNV is necessary for further interpretation of prenatal results. Prenatal samples of 370 patients (Figure 1) with indications for chromosome studies were tested using whole-genome aCGH analysis at the Prenatal Diagnostic Laboratory, Tsan Yuk Hospital. Of those 370 prenatal samples, 220 patients had been prospectively recruited for the first-tier test study prior to the invasive procedure. In addition, 150 subjects with abnormal fetal ultrasound findings were retrospectively recruited into the ‘further-test’ study once conventional cytogenetic results had shown a normal karyotype on invasive testing. A total of 193 parental blood samples were used to categorize unclear CNVs identified in the corresponding prenatal samples in the evaluation study. In addition to the evaluation study, abnormal findings of 12 prenatal samples tested by conventional cytogenetics and requiring characterization were assessed using aCGH.

Conventional cytogenetics was performed by Giemsa banded (G-banded) karyotyping as a clinical service on all prenatal samples at Prenatal Diagnostic Laboratory, Tsan Yuk Hospital. As per protocol, optimally, 3–5 mg of dissected chorionic villi or 30 ml amniotic fluid was obtained to set up for karyotype, QF-PCR and aCGH. Cultured cells in flasks were used when either a) there were not enough cells in the primary sample, or b) in retrospective samples for the ‘further-test’ study, or c) in retrospective samples required for characterization studies. Eleven placental tissue samples and 2 skin biopsy samples obtained from pregnancies which were terminated after abnormal fetal ultrasound findings were processed for aCGH analysis.

### Indications for recruitment to the study

Prenatal patients with clinical indications for further diagnoses were recruited to the research study. The reasons included abnormal findings on fetal ultrasound; positive Down syndrome screening; or maternal anxiety concerning advanced maternal age, family history of genetic disorder or previous child with anomalies. Some patients met more than one of the indications for study. In categorizing the indications as shown in Figure 1, ultrasound abnormality preceded positive Down syndrome screening and in turn over maternal anxiety.

### Methods of DNA extraction

Cells were pelleted from 5 ml amniotic fluid by centrifugation at 400×g for 10 min. DNA was extracted by Gentra Puregene Tissue Kit (QIAGEN, USA) following manufacturer’s instruction. Uncultured chorionic villi, tissue or cultured cells were pelleted by centrifugation at 3000×g for 5 min, lysed in 300 µl of lysis buffer (100 mM Tris, pH 8.5, 5 mM EDTA, 0.2% SDS, 200 mM NaCl) with Proteinase K at final concentration of 2 mg/ml. The lysate was incubated at 55°C overnight, added with 7 µl of RNase A solution (Qiagen, USA) and incubated at 37°C for 60 to 120 min. DNA was precipitated by the addition of 2.5 volume of cold 100% ethanol, spooled, washed twice with 1 ml of 70% ethanol and air dried. The DNA pellet was dissolved in Tris EDTA (TE) buffer (10 mM Tris, 0.1 mM EDTA, pH 7.5). For the blood samples, 3 ml of EDTA blood were diluted to 9 ml with 1X PBS. The diluted blood was overlaid onto 6 ml of Ficoll-Paque Plus (GE Healthcare Life Sciences) and centrifuged at 600×g for 30 min. The mononuclear cells at the interphase were transferred to a fresh tube and washed twice with 15 ml of 1X PBS. Cells were pelleted by centrifugation at 400×g for 10 min. The cells were lysed in 1 ml of lysis buffer (100 mM Tris, pH 8.5, 5 mM EDTA, 0.2% SDS, 200 mM NaCl) and DNA was precipitated and processed as above. Where DNA extraction was deemed urgent, for example in advanced gestations, a commercial kit (QIAmp DNA blood kit, Qiagen, USA) was used. The concentration of DNA samples was measured by NanoDropND-1000 spectrophotometer (NanoDrop Technologies, USA) and the quality of DNA samples was analyzed by agarose gel electrophoresis to exclude degradation or RNA contamination.

### Array CGH analysis and interpretation

All samples were tested by NimbleGen CGX-135K arrays which were designed by Signature Genomics (Perkin Elmer, USA) following manufacturer’s instructions. The coverage of the array has an average resolution of 140 kb across the genome and 40 kb or less in regions of clinical relevance. It evaluates over 245 known genetic syndromes and over 980 gene regions of functional significance in human development. The data were analyzed by Genoglyphix software (Signature Genomics, Spokane, USA). The gender of the prenatal samples was examined using QF-PCR [14] to determine the gender-matched reference DNA used in aCGH. The control DNA was from pooled gender-matched DNA from Promega (Madison, Wisconsin, USA).

Copy number variants (CNVs) detected by aCGH were systematically evaluated for clinical significance by comparison with information in the Signature Genomics’ proprietary Genoglyphix Chromosome Aberration Database (Signature Genomics, Spokane, WA, USA), the internal laboratory database at Tsan Yuk Hospital, and the publicly available databases [Database of Genomic Variant (DGV), International Standards for Cytogenomic Arrays Consortium Database (ISCA), Children Hospital of Philadelphia database (CHOP), Database of Chromosomal Imbalance and Phenotype in Humans using Ensembl Resources (DECIPHER)]. Categorization of CNVs was based on available information concerning the fetal phenotypes and by comparison of phenotypes with known genes in the region of copy gain or loss. This was ascertained from searching Online Mendelian Inheritance in Man (OMIM), PubMed, RefSeq and the University of California Santa Cruz (UCSC) genome browser. A CNV was considered to be: (1) benign if it was reported in healthy subjects in the databases searched; if there are no genes involved; or if involved genes were unrelated to the phenotype and have no apparent clinical relevance; (2) clinically significant if it corresponded to a region known to be of clinical relevance or had a gene of clinical relevance; (3) of unclear clinical significance if there is insufficient evidence to categorize as clinically significant or benign at the time of reporting. When CNVs of unclear clinical significance were detected in a prenatal sample, parental blood samples were processed to provide additional information for interpretation.

### Confirmation of CNVs

Clinically significant copy number gains and losses not detectable by karyotyping were confirmed by Fluorescent In Situ Hybridization (FISH) studies whenever possible. Microdeletion and telomeric FISH probes were obtained from Abbott Diagnostics (USA) and FISH probes from bacterial artificial chromosome clones from The Centre for Applied Genomics at the Hospital for Sick Children (Toronto, Canada). Homozygous alpha thalassemia deletion was confirmed by standard laboratory protocol [15].

Microarray data are available in the ArrayExpress database (www.ebi.ac.uk/arrayexpress) under accession number E-MTAB-2156.

## Results

Clinically significant CNVs identified by aCGH in the prenatal samples from both the first-tier test study and from the ‘further-test’ study are shown in Figure 1. The initial turn-around time for aCGH was 8–10 days, while conventional cytogenetics took 14–21 days (data not shown).

### First-tier test study

Two hundred and twenty prenatal samples were examined using whole-genome aCGH methodology together with routine culture for G-banded karyotyping with or without QF-PCR rapid aneuploidy testing. One hundred and sixteen samples (52.7%) were positive for Down syndrome screening with no apparent fetal ultrasound anomalies at the time of testing; whilst 77 prenatal samples (35.0%) had fetal ultrasound anomalies detected prior to invasive prenatal diagnosis. Forty of these fetal malformations concerned a single organ system and 37 had abnormalities involving more than one organ system. In 27 prenatal samples (12.3%), maternal anxiety was the clinical indication for invasive testing and inclusion into the study (Figure 1).

Clinically significant CNVs were detected in 44 (20%) out of 220 samples (Table 1). Common aneuploidies were detected in 21 prenatal samples (9.5%) with six cases of trisomy 21; seven cases of trisomy 18; four cases of trisomy 13; and four cases of monosomy X. Twenty three prenatal samples (10.5%) had other chromosomal imbalances, as summarized in Table 2, 3, 4. There were 7 (3.2%) prenatal samples with CNVs detected by aCGH which had been undetected by karyotyping (Table 2, Case no. 1–7). In 9 (4.1%) samples, aCGH revealed additional information over G-banded karyotyping (Table 3, Case no. 8–16). These included 4 complex chromosomal rearrangements (Table 3, Case no. 8–11) involving 2 or more chromosome segments not attributed to unbalanced translocations, one additional marker chromosome (Table 3, Case no. 12), 2 additional ring structures (Table 3, Case no. 13 and 14), one mosaic ring chromosome 18 (Table 3, Case no. 15) and one unbalanced translocation which aCGH helped to define a small deletion in the derivative chromosome (Table 3, Case no. 16). In 7 samples (3.2%), aberrations were detected both by aCGH and karyotyping. These included 4 unbalanced translocations (Table 4, Case no. 17–20), trisomies involving chromosome 7 or 16 (Table 4, Case no. 21, 22) and one case with terminal deletion of chromosome 13 was identified (Table 4, Case no. 23). The result of the first-tier study showed an additional diagnostic yield of 3.2% (7/220) for aCGH over conventional G-banded karyotyping. One triploidy was not detected by aCGH.

**Table 1:** Clinically significant CNVs detected in the first-tier test study.

		Clinically significant CNV*		
Indication	Samples	Common aneuploidies (%)	Other abnormalities (%)	Total (%)
DS positive (no USS abn)	116	3 (2.6)	3 (2.6)	6 (5.2)
USS abn	77	18 (23.4)	20 (26.0)	38 (49.4)
Anxiety	27	0	0	0
Total	220	21 (9.5)	23 (10.5)	44 (20.0)

DS: Down syndrome screening; USS abn: ultrasound abnormality;

*percentage of clinical significant CNV found in the indication category.

**Table 2:** Clinically significant CNVs other than common aneuploidies detected in the first-tier test study which were not detected by karyotyping.

Case No.	Gest (w)	Sample type	Indication for study	Initial Karyotype	Array results	CNV size and type/syndrome or locus	Outcome	Phenotype and other information
1	18+6	AF	USS: ventriculomegaly, partial agenesis of corpus callosum	46,XY	arr 1q43q44(236,811,017–247,174,728)×1 dn	10.36 Mb loss/1q44 microdeletion syndrome	TOP at 22 w	Postmortem: agenesis of corpus callosum. Updated karyotype: 46,XY,del(1)(q43)
2	22	Placental Tissue	USS: hypoplastic tibiae and fibulae	46,XX	arr 19p13.3q13.43(213,080–63,703,259)×2∼3 dn	mos +19	TOP at 21 w	Clinical diagnosis: brachyphalangy, polydactyly and tibial aplasia/hypoplasia syndrome. Updated karyotype: mos 47,XX,+19 [6]/46,XX [94]
3	16+4	Cultured AF	DS +ve, USS: early onset IUGR, fetal heart in right thorax, NT 4.6 mm in first trimester	46,XX	arr 2p25.3q11.2(26,551–100,807,481)×2∼3 dn	mos 100.78 Mb gain/mos +2p	TOP	Postmortem: fetal heart in right thorax, diaphragmatic hernia, left lung hypoplasia, ASD, 1A1V.Updated karyotype: mos 47,XX,+del(2)(q11.2) [3]/46,XX [27]
4	17+5	AF	Alpha thalassaemia couple, USS: increased cardio-thoracic ratio	46,X,inv(X)(p21q22.1)	arr Xq22.1(99,482,878-99,663,419)×3 dn,16p13.3(162,893-169,832)×0	217 kb gain at Xp21.1, 180 kb gain at Xq22.1/Unclear epilepsy & Intellectual disability restricted to female, 6.94 kb 2-copy loss at 16p13.3/Hb Bart's disease	TOP at 18 w	Hb Bart's disease
5	24	AF	USS: holoprosencephaly, proboscis, hypotelorism	46,XY	arr 8q23.3q24.12(113,718,332-122,076,557) ×1 dn	8.36 Mb loss in 8q23.3-8q24.12/Langer-Giedion/Trichorhinophalangeal	TOP at 23w	No information
6	20+2	AF	USS: Bilateral hand deformity, missing middle finger, compatible with ectrodactyly	46,XX	arr 17p13.3(1,059,027-1,218,853)×4 mat,17p13.3(2,260,944-2,366,399)×4 mat	Two copy gain 159.83 kb gain in 17p13.3	TOP at 23 w	Postmortem: bilateral ectrodactyly
7	13+4	CV	USS: enlarged fetal bladder 1.03×1.08×0.8 cm. Dilated renal pelvis	46,XY	arr 16q24.1(83,708,997-85,428,578)×1 dn	1.72 Mb loss	TOP at 19+3 w	Postmortem: bilateral low set ears, urethral atresia, bilateral hydroureters and renal dysplasia, malrotation of gastrointestinal tract, rectal atresia

AF: amniotic fluid; ASD: atrial septal defect; CV: chorionic villi; DCDA: dichorionic diamniotic; DS: Down syndrome screening; FB: fetal blood; Gest: Gestation; IUGR: intrauterine growth restriction; PPROM: preterm premature rupture of membranes; TOP: termination of pregnancy; USS: ultrasound scan findings; w: weeks; +ve: positive.

**Table 3:** Clinically significant CNVs other than common aneuploidies detected in the first-tier test study with additional information provided by aCGH over karyotyping.

Case No.	Gest (w)	Sample type	Indication for study	Initial Karyotype	Array results	CNV size and type/syndrome or locus	Outcome	Phenotype and other information
8	32	Cultured AF	USS: fetal hydrops, pleural effusion, lung hypoplasia, ascites, bilateral hydrocele, polyhydramnios	46,XY,15q+dn	arr 15q11.2q13.2(20,372,901-28,138,979)×4 dn,15q13.2q13.3(28,927,707-30,226,405) ×3 dn	7.77 Mb 2-copy gain/15q11-q13 Microduplication region 1.3 Mb gain/15q13.3 Microdeletion region	Stillborn at 32+2w	Postmortem: hydrops fetalis with severe interstitial edema and hypoplastic lungs
9	17	CV	Maternal age, USS: DCDA twins - one IUGR, the other reduced because of acrania	46,XX,16p-	arr 16p13.3p13.2(2,912,285-7,697,824)×1 dn, 16p13.2(7,702,318-8,933,435)×1∼2 dn, 16p13.13(11,323,756-12,017,936)×2∼3 dn	4.79 Mb loss/16p13.3 Microdeletion-Severe Rubinstein-Taybi syndrome, 1.23 Mb mosaic loss, 694.18 kb mosaic gain	TOP at 19w	Postmortem: hypertelorism with prominent slanting of upper and lower inner epicanthic folds, wide mouth and roundish receding chin, pre-axial polydactyly of right hand, 11 pairs of ribs, ventricular septal defect, right renal and right ovarian agenesis, single umbilical artery
10	35+4	Cultured AF	DS -ve, USS: strawberry head, absent vermis, short long bones, abnormal heart	47,XY,+mar dn	arr 9p24.3p23(199,254-24,838,669)×4, 9p21.3p21.1(24,871,570-38,751,949)×3	24.6 Mb 2-copy gain, 13.87 Mb gain/+9p	TOP	Baby abnormal
11	22+4	Placental Tissue	USS: truncus arteriosus, dilated cerebral ventricles, hydronephrosis, absent nasal bone	46,XX,der(8)(qter–>q21.3::p23–>qter)dn	arr 8p23.3p23.1(192,262-10,096,394)×1 dn, 8p23.1(10,620,658-11,895,875)×4 dn, 8q21.3q24.3(91,500,429-146,264,292)×3 dn	9.9 Mb loss/8p23.1 microdeletion(CDH2) syndrome, 1.28 Mb 2-copy gain/8p23.1 microduplication syndrome, 54.76 Mb gain	TOP at 22+4w	Postmortem: hypertelorism, flattened nasal bridge, down slanting palpebral fissure, micrognathia, high arched palate, midline alveolar cleft, truncus arteriosus type I with ventricular septal defect, atretic left pulmonary trunk origin, mild thymic hypoplasia, hydrocephalus with dilated lateral ventricles and fourth venticle and dilated renal pelves
12	10+3	Cultured CV	Maternal age, USS: mild ventriculomegaly 11-12 mm, micrognathia	47,XX,+mar	arr 9p24.3q22.31(199,254-94,915,798)×3	94.72 Mb gain	TOP	Updated karyotype: 47,XX,+del(9)(q22)
13	35	Fetal blood	IUGR	46,XX [27]/47,XX,+mar dn [79]	arr 18q11.1q21.32(16,796,771-54,412,918)×3	37.62 Mb gain	TOP	Updated karyotype: mos 46,XX/47,XX,+r(18)
14	16+6	Cultured AF	DS +ve, CVS: mos 47,XX,+mar [8]/46,XX [22]	mos 47,XX,+mar dn [20]/46,XX [20]	arr 1p13.1p12(116,138,882-120,311,704)×2∼3 dn	4.17 Mb gain	TOP	Abortus: facial asymmetry with hypoplasia of the left face and jaw, club feet; Updated karyotype: mos 47,XX,+r(1)dn [20]/46,XX [20]
15	20+3	Cultured AF	DS +ve, CVS: mosaicism with 3 cell lines: 45,XY,-18 (36.67%), 46,XY,r(18)(p11.3q23)(63.33%), 47,XY,r(18)×2(3.33%)	mos 45,XY,-18 [9]/46,XY,r(18)(p11.3q23) [27]	arr 18p11.32p11.21(131,491-14,107,537)×1, 18q11.1q23(16,796,771-74,503,562)×1∼2, 18q23(74,508,960-76,114,684)×1	13.98 Mb loss in 18p11.32p11.21, mos 57.71 Mb loss at 18q11.1q23, 1.61 Mb loss at 18q23	TOP at 20w	Postmortem: low set ears
16	23	AF	USS: thickened myocardium with thin rim of pericardial effusion	46,XY,18q+	arr 2p25.3p23.2(26,551-27,758,445)×3, 18q23(75,373,273-76,114,684)×1	27.73 Mb gain at 2p, 741.41 kb loss at 18q	TOP at 23w	Postmortem: patent ductus arteriosus. Updated karyotype: 46,XY,der(18)t(2;18) (p23;q23)pat

AF: amniotic fluid; ASD: atrial septal defect; CV: chorionic villi; DCDA: dichorionic diamniotic; DS: Down syndrome screening; FB: fetal blood; Gest: Gestation; IUGR: intrauterine growth restriction; PPROM: preterm premature rupture of membranes; TOP: termination of pregnancy; USS: ultrasound scan findings; w: weeks; +ve: positive.

**Table 4:** Clinically significant CNVs other than common aneuploidies detected in the first-tier test study with abnormalities detected by both aCGH and karyotyping.

Case No.	Gest (w)	Sample type	Indication for study	Initial Karyotype	Array results	CNV size and type/syndrome or locus	Outcome	Phenotype and other information
17	23+3	Cultured AF	USS: diaphragmatic hernia, hyperextended right knee	46,XX,der(15)t(4;15)(p15.2;q26.1)pat	arr 4p16.3p15.1(33,860-28,155,263)×3 dn, 15q26.1q26.3(87,502,509-100,208,480)×1 dn	28.12 Mb gain at 4p, 12.71 Mb loss at 15q/Congenital diaphragmatic hernia	TOP at 23w	Postmortem: low set ears, receded chin, clenched fists, right knee dislocation, rocker-bottom feet, left diaphragmatic hernia, lung hypoplasia
18	11+2	CV	USS: cystic hygroma	46,XX,der(7)t(7;11)(q35;p12)pat	arr 7q35q36.3(146,772,782-158,816,094)×1, 11p15.5p12(195,983-42,558,628)×3	12.04 Mb loss at 7q, 42.36 Mb gain at 11p	Miscarriage at 16w	Abortus: cleft lip
19	13+1	CV	USS: cardiomegaly, club feet at 13w. No obvious fetal abnormalities seen at 16w. Previous child with 46,XX,t(5;9)(p15.2;p21)mat	46,XY,der(5)t(5;9)(p15.2;p21)mat	arr 5p15.33p15.2(108,467-13,978,254)×1, 9p24.3p21.1(199,254-29,362,821)×3	13.87 Mb loss in 5p, 29.16 Mb gain in 9p	TOP at 16w	Postmortem: no anatomical abnormality
20	21+6	AF	USS: bilateral complete cleft lip and bilateral cleft palate	46,XX,4p-	arr 4p16.3p16.1(33,860-8,772,114)×1,8p23.3p23.1(192,262-6,907,722)×3	8.74 Mb loss in 4p/Wolf Hirschhorn syndrome, 6.72 Mb gain in 8p	TOP	Postmortem: bilateral cleft lip and palate. Updated karyotype: 46,XY,der(4)t(4;8)(p16;p23)dn
21	12+6	CV	Maternal age, DS +ve	47,XX,+7	arr 7p22.3q36.3(136,363-158,816,094)×2∼3	mos +7 (60%)	Cervical incompetence; spontaneous miscarriage at 21w	Postmortem: single umbilical artery
22	14+2	Placental tissue	DS +ve, USS: mild cardiomegaly, pericardial effusion, increased placental thickness	mos 47,XX,+16 [8]/46,XX [14]	arr 12q22q23.1(93,607,866-95,066,901)×3 Trisomy 16	+16	PPROM at 14w and TOP performed	No information
23	22+5	AF	USS: abnormal brain with echoic shadow 3.9 cm, ? absent corpus callosum, holoprosencephaly	46,XY,13q-	arr 13q32.1q34(96,486,944-114,109,838)×1	17.62 Mb loss in 13q32.1-13q34	TOP at 23w	Postmortem: holoprosencephaly, ASD. Updated karyotype: 46,XY,del(13)(q32)dn

AF: amniotic fluid; ASD: atrial septal defect; CV: chorionic villi; DCDA: dichorionic diamniotic; DS: Down syndrome screening; FB: fetal blood; Gest: Gestation; IUGR: intrauterine growth restriction; PPROM: preterm premature rupture of membranes; TOP: termination of pregnancy; USS: ultrasound scan findings; w: weeks; +ve: positive.

The clinically significant results were analyzed and categorized according to the clinical indications for the investigations. There were six cases of patients who had screened positive for Down syndrome without fetal ultrasound anomalies being evident (Table 1). Three cases had common aneuploidies (one each of trisomy 13, 18, 21) and the remaining three had other chromosomal imbalances including ring structures and trisomy 7 (Table 3, Case no. 14, 15, and Table 4 Case no. 21). In 77 prospective samples with fetal ultrasound anomalies detected (Table 1), 38 (49.4%) showed clinically significant chromosomal imbalances. Of these, 18 were common aneuploidies (three trisomy 13, six trisomy 18, five trisomy 21 and four monosomy X). In addition, 20 had other chromosomal imbalances (Table 2, Case no. 1–7, Table 3, Case no. 8–13, 16, Table 4, Case no. 17–20, 22 and 23). Eleven out of the 20 cases had fetal ultrasound anomalies in more than one organ system (including the neck and body fluid, central nervous system, cardiovascular, craniofacial, gastrointestinal, genitourinary, musculoskeletal, thoracic, or other anomalies including intrauterine growth restriction). Where invasive testing was indicated for maternal anxiety, no chromosomal imbalance was detected in the 27 samples studied.

### Further-test study after normal karyotyping

In 150 patients with normal karyotyping and abnormal fetal ultrasound findings, nine clinically significant CNVs were identified using aCGH. All of the patients had fetal ultrasound detected anomalies in more than one organ system (Table 5). These included one 1p32 microdeletion resulting in *NFIA* haploinsufficiency [16]; one 22q11.2 microdeletion, one deletion in chromosome 14 resulting in paternal uniparental disomy 14-like phenotype; one 8p23.1 microdeletion; one unbalanced translocation detected after an apparent normal karyotype and four samples with microdeletion in 16p13.3 resulting in Hemoglobin Bart’s disease. With the exception of the unbalanced translocation which was undetected on chorionic villus karyotyping, but identified by aCGH of the amniotic fluid and cytogenetic study, the remaining CNVs detected were <5 Mb in size and submicroscopic, beyond the detection resolution of conventional karyotyping. The additional diagnostic yield of aCGH over conventional cytogenetics was found to be 6%.

**Table 5:** Overview of the clinical aspects of the fetuses with clinically significant CNVs detected in the further-test study with normal karyotype.

Case No.	Gest (w)	Sample type	DS +ve	Ultrasound abnormalities	Karyotype	Array results	CNV size and type	Locus/syndrome	Outcome	Phenotype and other information
1	23+4	AF	Y	Absent corpus callosum, prominent posterior horn of lateral ventricle. MRI fetal brain: Absent cerebellar vermis, complete absence of corpus callosum	46,XX	arr 1p32.1p31.3(60,638,478-64,100,969)×1 dn	3.46 Mb loss	*NFIA* haplo-insufficiency	TOP at 23+4w	Postmortem: absence of cerebellar vermis and corpus callosum
2	21	AF	N	Left axis deviation, TOF with VSD, over-riding aorta, small PA	46,XY	arr 22q11.21(17,299,469-19,790,658)×1	2.49 Mb loss	22q11.2 microdeletion	TOP at 22w	Postmortem: TOF
3	33	AF	N	Polyhydramnios, short limbs, small stomach bubble	46,XY	arr 14q32.2q32.31(99,890,594-101,081,825)×1 mat	1.19 Mb loss	UPD(14)pat-like phenotype	Live birth at 34w, 2.41 kg	Newborn with pharyngolaryngomalacia and obstructive sleep apnoea syndrome
4	12+2	CV	Y	Cystic hygroma, subcutaneous oedema, exomphalos	46,XY	arr 8p23.1(8,146,273-11,895,875)×1	3.75 Mb loss	8p23.1 Microdeletion	Live birth at 39w, 2.96 kg	Secundum ASD.
5	12+	CV	N	Skin oedema and NT 6 mm	46,XY	arr 10p15.3p13(128,680-15,889,188)×3,13q33.1q34(102,510,400-114,109,838)×1	15.76 Mb gain in 10p; 11.60 Mb loss in 13q	Unbalanced translocation (10;13)	TOP at 23w	USS at 21w: bilateral pleural effusion and ascites, amniocentesis performed for aCGH. Missed karyotype in CV, detected after aCGH in AF. Postmortem: hydrops fetalis (bilateral pleural effusion and ascites), polydactyly of right hand. Updated karyotype: 46,XY,der(13)t(10;13)(p13;q33)pat
6	13	CV	N	Fetal cardiomegaly, placentomegaly	46,XX	arr 16p13.3(162,893-169,832)×0	6.94 kb 2-copy loss	Hb Bart's disease	TOP at 14w	No information
7	16+2	AF	N	Placenta thickened, increased CT ratio, abnormal hands+feet	46,XX	arr 16p13.3(162,893-169,832)×0	6.94 kb 2-copy loss	Hb Bart's disease	TOP at 17w	Abnormal hands & feet, cardiac hypertrophy
8	13+1	CV	N	Placentomegaly, cardiomegaly	46,XX	arr 16p13.3(162,893-169,832)×0	6.94 kb 2-copy loss	Hb Bart's disease	TOP at 14w	Cardiomegaly
9	32	FB	N	USS: fetal hydrops with ascites & cardiomegaly, dilated RA, left to right shunt, MCA PSV normal	46,XX	arr 16p13.3(162,893-169,832)×0	6.94 kb 2-copy loss	Hb Bart's disease	Emergency Caesarean section at 32+1w, 1.48 kg, NND	Postmortem: ASD and pulmonary hypoplasia, placental hydrops

AF: amniotic fluid; ASD: atrial septal defect; CT: cardio-thoracic; CV: chorionic villi; DORV: double outlet right ventricle; DS: Down syndrome screening; FB: fetal blood; FU: follow up; Gest: Gestation; Hb: haemoglobin; N: not DS +ve or not mentioned; NND: neonatal death; PA: pulmonary artery; PS: pulmonary stenosis; TOF: Tetralogy of Fallot; TOP: termination of pregnancy; USS: ultrasound: VSD: ventricular septal defect; w: weeks; Y:yes; +ve: positive.

### CNV of unclear clinical significance

The detection rate for CNVs of unclear significance identified during the evaluation study using the 135 K whole-genome array was 2.7% (10/370), with 1.4% (3/220) detected for the first-tier test and 4.7% (7/150) for ‘further-test’ (Table 6). Six of these 10 samples had CNVs associated with microdeletions or microduplications with incomplete penetrance and variable expressivity (Table 6, Case no. 1, 2, 4–7). Eight samples involved genes or loci which may be associated with neurodevelopmental problems (Table 6, Case no. 1–8). Two samples involved genes associated with structural abnormalities of organ systems that may or may not have relevance to the phenotype (Table 6, Case no. 7, 8). One de novo microdeletion of 1q21.1 with susceptibility for thrombocytopenia-absent radius (TAR) was classified as a CNV of unclear significance having determined that the fetal radii were present on the follow-up ultrasound scan (Table 6, Case no. 7). Where CNVs of unclear clinical significance were identified, the incidence of de novo CNVs was found to be 0.9% of the total in the study (3/370).

**Table 6:** Overview of the clinical aspects of the fetuses with CNVs of unclear clinical significance.

Category	Case No.	Gest (w)	Sample type	DS +ve	Ultrasound abnormalities	Karyo-type	Array results	CNV size and type	OMIM Genes/Locus	Outcome	Phenotype and other information
First-tier test	1	19+2	AF	Y	DS+ve risk 1∶105	46,XX	arr 1q21.1(144,998,070-146,193,043)×1 mat	1.19 Mb loss	1q21.1 microdeletion	Live birth at 37+6w, 3.14 kg	No abnormality at birth. FU paediatricians for failure to thrive. Growth parameters below 3rd centile.
	2	20	AF	N	USS: early onset IUGR	46,XX	arr 16p11.2(29,564,890-30,100,123)×1 dn	535.23 kb loss	16p11.2 microdeletion	TOP at 24w	No information
	3	13+5	CV	Y	DS +ve 1st tri, risk 1∶190, USS: increased NT 4.1 mm	46,XY	arr 3p26.3(76,277-3,092,911)×1 pat,9p24.3(485,809-551,031)×1 pat	3.02 Mb loss at 3p, 65.22 kb loss at 9p	*CHL1, CNTN6, CNTN4, IL5RA, KANK1*	Live birth at 39+5w, 3.92 kg	No abnormality at birth. Last update of baby normal.
Further test	4	12+3	Cultured CV	Y	USS: 12wk scan showed cystic hygroma, NT 5.6 mm, 18wk scan showed TOF	46,XY	arr 16p12.1(21,857,845-22,336,067)×1 dn	478.22 kb loss	16p12.1 microdeletion	TOP at 20w	Postmortem: TOF
	5	22+1	Cultured AF	N	USS: increased cisterna magnum 1.03 cm	46,XY	arr 16p13.11p12.3(15,419,888-18,054,322)×3 mat	2.63 Mb gain	16p13.11 microduplication	Live birth at 38+6w, 3.32 kg	No abnormality at birth.
	6	17+2	Cultured AF	N	USS: Increase NT 5.2 mm in 1st trimester. Previous child with bilateral SVC, dysmorphism, global developmental delay	46,XY	arr 15q11.2(20,372,901-20,636,841)×1 pat	263.94 kb loss	15q11.2 microdeletion	Live birth at 40+6w, 2.84 kg	Noted bilateral preauricular sinuses at birth. FU paediatricians for 15q11.2 microdeletion from paternal origin (tested at another unit). Last update of baby normal.
	7	22+5	Cultured AF	N	USS: TOF, small thymus	46,XX	arr 1q21.1(144,100,334-144,458,066)×1 dn	357.73 kb loss	1q21.1 microdeletion with susceptibility for thrombocytopenia-absent radius (TAR)	Live birth at 38w, 2.22 kg.	Last FU at 9 months: 6 kg. TOF with surgical correction done; left indirect inguinal hernia with herniotomy done; clefting of soft palate; poor feeding with recurrent projectile vomiting; failure to thrive with short stature; insucking of chest since birth
	8	21+6	Cultured AF	N	USS: TGA	46,XX	arr 2q13(111,114,738-112,817,963)×1 mat	1.7 Mb loss	*BUB1, BCL2L11, ANAPC1, MERTK, FBLN7*	TOP at 23+6w	Postmortem: TGA
	9	19	Cultured AF	N	USS: large omphalocoele with liver herniation, TOF	46,XY	arr 2q21.1(130,769,854-131,199,432)×3 pat	429.6 kb gain	*CCDC115, IMP4, PTPN18, CFC1*	TOP at 23+2w	Postmortem: omphalocoele, TOF, single umbilical artery
	10	22	Cultured AF	N	USS: bilateral club feet. Right hand held in fixed flexion position with overlapping finger	46,XX	arr 2p21(45,025,361-45,129,076)×3 pat,15q13.3q14(30,846,564-31,432,930)×3 mat	98.46 kb gain, 586.4 kb gain	*SIX3, SIX2, FMN1, RYR3*	TOP at 23+4w	Postmortem: low set ears, multiple joint contractures, compatible with arthrogryposis multiplex congenita

AF: amniotic fluid; CM: cisterna magna; CV: chorionic villi; DS: Down syndrome screening; FU: follow up; Gest: Gestation; IUGR: intrauterine growth restriction; N: not DS +ve or not mentioned; NT: nuchal translucency; SVC: superior vena cava; TGA: transposition of great arteries; TOF: Tetralogy of Fallot; TOP: termination of pregnancy; USS: ultrasound; Y: yes; +ve: positive.

### Characterization by aCGH on samples with abnormal karyotypes

During the study period, in addition to the 370 samples for the evaluation study, twelve prenatal samples were identified with abnormal karyotypes during conventional cytogenetics. These were further examined by aCGH. Breakpoints were established for two samples with unbalanced translocations and three samples with interstitial deletions. One sample used aCGH to confirm suspected deletion of chromosome 16 pter which had been undetermined by G-banding. This sample was found to have a balanced translocation between chromosome 2 and chromosome 10 which was inherited from the father. It was reassuring that no submicroscopic changes were evident. Three samples showed complex rearrangements, one involved a marker chromosome derived from chromosome X, one involved a marker chromosome derived from chromosome 15 which had the same array findings as Case no. 8 in Table 3. One complex chromosomal rearrangement involved translocation of a 1.76 Mb segment from chromosome 19 to a duplicated segment of chromosome 2. These three complex rearrangements would not have been accurately detected by conventional karyotyping.

### Common benign CNVs

A total of 563 samples (370 prenatal and 193 parental) were performed using aCGH for the first-tier test, ‘further-test’ studies and for abnormal chromosomal characterization. These studies established common benign CNVs at eleven loci in patients of Hong Kong (Table 7). The most common locus with chromosomal gain or loss found in Hong Kong was at 8p11.23, and was detected at a frequency of up to 78% (Table 7, no. 5) reflecting the homogeneity of the local population. The proportion of parental samples required to be performed was 144 out of 193 (74.6%) in the first half period of evaluation and 49 out of 193 (25.4%) in the second half. The knowledge of common benign CNVs in the local population reduced the need for parental sample testing and could minimize cost if aCGH use in prenatal diagnosis is launched as clinical service.

**Table 7:** Common benign CNVs found in Hong Kong.

No.	Region size (Kb)	Cytoband Location	Genome coordinates	Event	Genes	Frequency of gain/loss (% )
1	194	1q31.1	chr1∶187592011–187776739	Loss	0	9
2	37	1q44	chr1∶246644054–246914515	Gain/Loss	1	29/13
3	122	6p25.3	chr6∶210793–321392	Gain/Loss	1	7/18
4	171	7p22.3	chr7∶136,363–325,833	Gain	0	14
5	97–125	8p11.23	chr8∶39310297–39531197	Gain/Loss	1–2	51/27
6	68–504	14q11.2	chr14∶21388121–22089869	Gain/Loss	0–2	23/12
7	3–180	16p12.1	chr16∶22534936–22689740	Gain/Loss	0	1/11
8	320	17q21.31	chr17∶41507230–42147712	Gain/Loss	1	2/55
9	83	19p12	chr19∶20408868–20518856	Loss	0	20
10	123	Xp22.33	chrX:3761569–3863478	Gain/Loss	1	9/34
11	105–109	Xq28	chrX:153064828–153168166	Gain/Loss	3	23/9

Kb: Kilobase.

### Increased nuchal translucency (NT)

Among all 382 prenatal samples, 27 of them had NT of 3.5 mm or above. In seven of these 27 prenatal samples Down syndrome screening results were not available. The remainders were screened positive for Down syndrome. There were three findings of trisomy 21, in addition to three samples with clinically significant CNVs detectable by karyotyping. There were three samples with CNVs of unclear clinical significance (Table 6, Case no. 3, 4, 6), one of which had structural abnormality on fetal ultrasound scan. Therefore aCGH did not yield increased detection of clinically significant CNVs compared to karyotyping in this specific number of samples with increased nuchal translucency.

## Discussion

This evaluation study demonstrated the effectiveness of the whole-genome oligonucleotide aCGH in prenatal diagnosis for the analysis of chromosome imbalance at high resolution. During the first-tier test, a detection rate of 20% was determined amongst patients with clinical indications for testing. Clinically significant imbalances were found to be common aneuploidies (9.5%), whilst 10.5% involved other chromosomal abnormalities (Table 1). Rapid aneuploidy testing such as QF-PCR is able to exclude common aneuploidies prior to testing using aCGH. This would make aCGH more cost effective. Scott *et al.* also proposed the use of combined QF-PCR and aCGH as first-line prenatal diagnostic testing [17]. Our detection rate of 10.5% clinically significant CNVs other than common aneuploidy was double that of Scott *et al.* (2013). It is considered that this may be related to different patient characteristics, different indications for prenatal testing, in addition to using whole-genome rather than targeted array. CNVs only detectable by aCGH were also higher in our evaluation study (3.2% in this study versus 1.2% pathogenic CNVs) [17].

Array CGH helped to precisely delineate breakpoints, characterize marker chromosomes and detect mosaicism within a shorter time frame compared to G-banded cytogenetics. There were five samples with chromosome mosaicism in the study. Mosaic trisomy 19 and mosaic trisomy 7 (Table 2, Case no. 2 and Table 4 Case no. 21) were results confined to placental mosaicism where studies on skin fibroblast and in the amniotic fluid sample respectively indicated normal karyotypes. Of interest was a mosaic ring chromosome 18 finding with aCGH of one copy number gain for the chromosome 18 segment (Table 3, Case no. 13), not matching the mosaic ratio, but the FISH study found a small number of double rings in the cell population which explained the array result.

**Figure 2:**
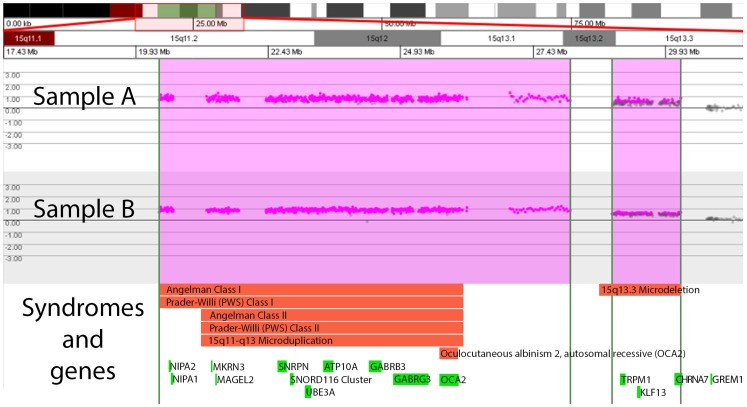
Identical complex chromosomal rearrangements in chromosome 15 found in 2 prenatal samples with different karyotypes and phenotypes. Sample A karyotype is 46,XY,15q+ dn (Table 3, Case no. 8); Sample B karyotype is 47,XX,+mar from characterization study. Each dot on the X-axis represents one oligonucleotide probe on the respective chromosome position. Two-copy gain is detected at 15q11.2q13.2 with minimum gain of 7.77 Mb. Single copy gain is detected at 15q13.2q13.3 with minimum gain of 1.3 Mb. No probe is located in the segment between the 2 regions of copy gains, therefore the exact number of copy gained is unknown in the segment. The genetic syndromes (red boxes) and genes (green boxes) in the region denoted by Signature Genomics Genoglyphix software are shown in the lower panel.

Four complex chromosomal rearrangements were determined in the first-tier test study, and three complex rearrangements were identified from prenatal samples with abnormal karyotypes requiring characterization. This highlighted the advantage of higher resolution aCGH in chromosome analysis. There were two samples with identical complex rearrangement comprising 2-copy gain at the Prader Willi/Angelman syndrome region (15q11.2–q13.2) and one copy gain at the 15q13.3 Microdeletion region (15q13.2–q13.3, Figure 2). Karyotyping of the first sample (Figure 2 Sample A; Table 3, Case no. 8) showed 15q+ while the chromosomal gain in the second sample (Figure 2 Sample B) performed using aCGH for characterization was in a marker chromosome. Although aCGH showed the same chromosomal imbalances, the phenotypes of the 2 samples were very different. In the latter sample choroid plexus cyst was the only anomaly. Since other CNVs detected in Case no. 8 (Table 3) were benign and apparently common in the local population, the severe phenotype may have been caused by small mutation in another chromosomal region or an interruption of the region 15q11.2–q13.2 by a complex rearrangement. The 15q proximal region is known for its instability due to the presence of repeating DNA elements [18], [19] which may give rise to the triplication from centromere to breakpoint (BP) 4 plus duplication from BP4 to BP5 in these samples. Further investigation into the association of phenotypes and the manifestation of the copy number gain and imprinting [20] can be made on similar samples with rearrangements in the region. The finding also demonstrated the advances of complementary use of both aCGH and karyotyping in analyzing samples with multiple significant CNVs.

There were 4 cases of Hemoglobin Bart’s disease with clinically significant aCGH findings in the ‘further-test’ study. While routine prenatal screening for thalassaemia by mean corpuscular volume (MCV) is offered in our locality, one case presented late in the third trimester (Table 5, Case no. 9) and was a result of non-paternity. This highlighted one potential use of the test to diagnose Hemoglobin Bart’s disease in circumstances of non-paternity [21].

Uncertain CNVs tend to be a concern for clinicians in counseling. Adequate pre and post-test information and counseling by trained counselors, and a team approach involving obstetricians, clinical geneticists and laboratory scientists in CNV interpretation is beneficial and is adopted in our setting. In this evaluation study, both CNVs of clinical significance and CNVs of unclear significance were reported to referring doctors. In the 10 cases with CNVs of unclear clinical significance, 5 of the pregnancies, all with major ultrasound abnormalities, were terminated (Table 6). Maternal cell contamination was not determined during the evaluation study. However the impact of contamination on the interpretation of prenatal microarray has been reported [22]. Taking this into account, our laboratory has adopted the policy to routinely exclude maternal cell contamination by examining STR markers of 13, 18 and 21 in fetal and maternal samples.

**Figure 3:**
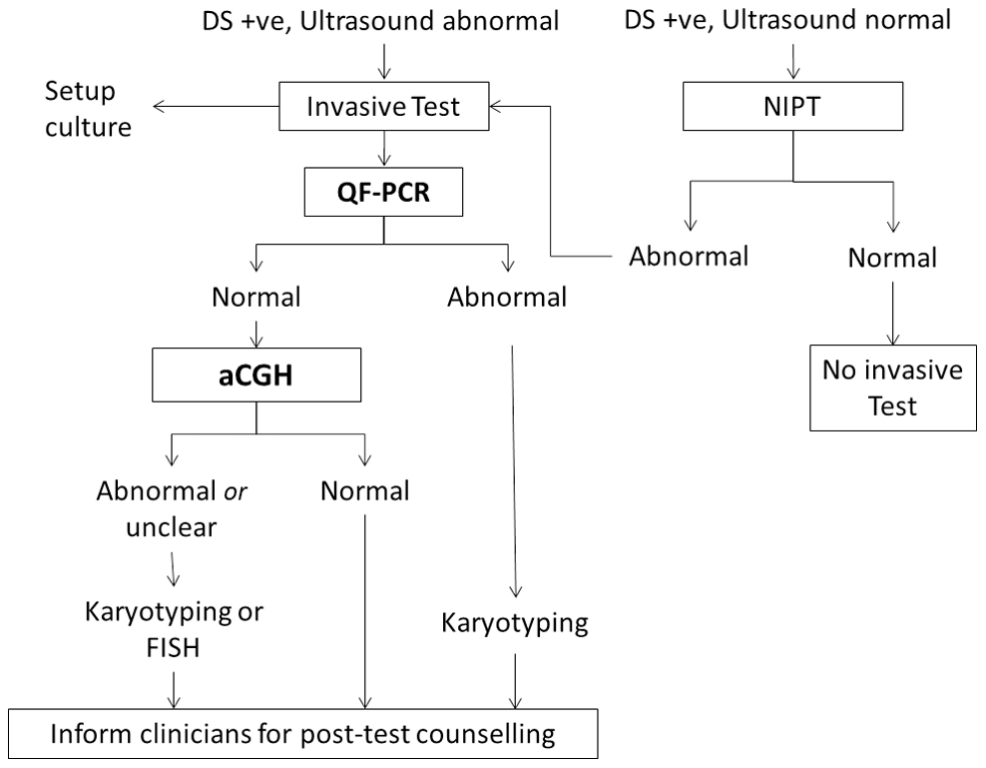
Proposed workflow for replacing karyotyping with aCGH in prenatal testing in Hong Kong. Pregnancies with Down syndrome screening positive without ultrasound abnormalities can be subjected to non-invasive prenatal testing; while pregnancies with Down syndrome screening positive in the presence of ultrasound abnormalities can be subjected to invasive test by QF-PCR to exclude common aneuploidy and maternal contamination, followed by aCGH as shown. aCGH, array CGH; DS+ve, Down syndrome screening positive; FISH, fluorescent in-situ hybridization; NIPT, non-invasive prenatal testing; QF-PCR, quantitative fluorescent-polymerase chain reaction for common aneuploidy detection.

Various authorities already approved the offering of aCGH as an adjunct diagnostic tool in prenatal cases with fetal ultrasound abnormalities [23]–[24]. The additional diagnostic yield of 6% in our further-test study is consistent with 5.2 to 10% increased detection by aCGH in fetuses with ultrasound abnormalities and normal karyotype reported in the literature [6], [7]. Data from a review study showed the overall detection rate of about 1% for significant submicroscopic aberrations in low risk pregnancies [10]. This consequently caused microarrays to be identified as a first-line diagnostic test in women choosing the test irrespective of clinical indication [9], [10], [25]. This is in line with the latest recommendations from the American College of Obstetricians and Gynecologists and the Society of Maternal-Fetal Medicine [26]. Based on these findings and reports in the literature [6]–[10], [25] on the potential of aCGH to detect chromosomal abnormalities beyond G-banded karyotyping, it is proposed that whole-genome aCGH would be suitable to replace conventional cytogenetics in prenatal diagnosis in Hong Kong (Figure 3). In a prenatal diagnostic setting, women screened positive for fetal Down syndrome may be offered an option of having noninvasive prenatal testing for fetal trisomy assessment by maternal plasma DNA if there is no ultrasound abnormality. In cases where ultrasound examination shows fetal abnormalities which require additional information from aCGH, an invasive diagnostic test could be performed. Rapid aneuploidy testing of the sample with QF-PCR would exclude common aneuploidies, triploidy and maternal contamination before aCGH analysis. This rapid test would identify triploidy which cannot be identified by aCGH. Cultures would be set up for cytogenetic study and karyotyping for all clinically significant CNVs (20.0%, Table 1) detected by aCGH if the CNV is large (>10 Mb). In cases where the CNV is small (<10 Mb), metaphase and interphase FISH could be performed. Conventional cytogenetics performed for abnormal QF-PCR findings (9.5% of samples, Table 1) would be needed to assess whether there is a parental balanced translocation carrier state in order to determine recurrence risk. This approach would reduce conventional cytogenetic testing by around 80% of the cases in Hong Kong. The disadvantages would include the potential for non-detection of balanced translocation or an inversion carrier status, low level mosaicism or small heterochromatic marker chromosomes of the fetus. Whilst non-detection of balanced translocation or inversion carrier status would be unlikely to affect the health of the fetus, low level mosaicism detection is a limitation of all prenatal investigation techniques. Further cost-benefit analysis and review of the staffing requirements of a cytogenetic laboratory may help to define the value of using microarrays for our prenatal diagnostic service provision.

NimbleGen has, however, phased out production of oligonucleotide 135 K arrays in favor of transitioning to Agilent oligonucleotide 8×60 K array, which has a lower backbone resolution. It is therefore anticipated that fewer CNVs of unclear significance will be detected for prenatal samples, with a shorter hybridization and faster turn-around time expected. Further studies will be required to confirm these effects.

## Conclusions

This evaluation study showed that the whole-genome 135 K aCGH platform increased the diagnostic yield of 3.2% using aCGH over conventional cytogenetics in the first-tier test study, and by 6.0% in the ‘further-test’ study for the Hong Kong population. It also offered a higher resolution karyotyping for prenatal diagnosis in both study models and results are comparable to recent published studies. It is proposed that aCGH should replace karyotyping for use in prenatal testing where invasive procedures are required, after excluding common aneuploidies and triploidies by quantitative fluorescent PCR. Conventional cytogenetics can be reserved for visualization of clinically significant CNVs.
